# Entropy of Muscle Fiber Histology Predicts Mobility in Older Adults: The Study of Muscle, Mobility, and Aging

**DOI:** 10.1111/acel.70421

**Published:** 2026-02-22

**Authors:** Namki Hong, Sang Wouk Cho, Alan A. Cohen, Russell T. Hepple, Paul M. Coen, Bumsoo Ahn, Anne B. Newman, Stephen B. Kritchesky, Paul J. Laurenti, Warren S. Browner, Steven R. Cummings

**Affiliations:** ^1^ San Francisco Coordinating Center California Pacific Medical Center Research Institute San Francisco CA USA; ^2^ Department of Epidemiology and Biostatistics University of California San Francisco CA USA; ^3^ Department of Internal Medicine, Institute of Endocrine Research Yonsei University College of Medicine Seoul South Korea; ^4^ Department of Biomedical Systems Informatics Yonsei University, College of Medicine Seoul South Korea; ^5^ Department of Environmental Health Sciences, Butler Columbia Aging Center, Mailman School of Public Health Columbia University NY USA; ^6^ Department of Physical Therapy University of Florida Gainesville FL USA; ^7^ AdventHealth Translational Research Institute Orlando FL USA; ^8^ Department of Internal Medicine Wake Forest University School of Medicine Winston‐Salem NC USA; ^9^ Department of Epidemiology, School of Public Health University of Pittsburgh PA USA; ^10^ Department of Internal Medicine, Section on Gerontology & Geriatric Medicine and the Sticht Center for Healthy Aging and Alzheimer's Prevention Wake Forest University School of Medicine Winston‐Salem NC USA; ^11^ Department of Radiology Wake Forest University School of Medicine Winston‐Salem NC USA

**Keywords:** aging, computer‐assisted, entropy, image processing, mobility limitation, muscles

## Abstract

Entropy may play an underappreciated role in human aging, such as in skeletal muscle functional declines. Histologically, muscle appears increasingly disorganized with aging, with greater fiber size variability and fiber‐type grouping. We tested the hypothesis that entropy is associated with reduced physical performance and muscle function, independent of muscle mass. We quantified a homeostatic dysregulation index of muscle (HDI_*M*_) as a proxy for entropy of muscle fiber disorganization based on cross‐sectional images of vastus lateralis biopsies from 299 adults age 70 or older. HDI_*M*_ was derived from three traits: fiber area diversity, fiber‐type heterogeneity, and the mean of the shortest path lengths through adjacent fiber networks. HDI_*M*_ derived from muscle fibers was highly correlated with Shannon entropy, a different measure of entropy of muscle fiber traits. Higher HDI_*M*_ derived from participants was associated with slower 400‐m walk speed, lower peak VO_2_, muscle power, and decreased maximum rate of oxidative phosphorylation by mitochondria in muscle. These findings suggest that muscle fibers accumulate entropy with aging which contributes to decline in physical performance, muscle power, and mitochondrial energetics, advancing the entropy framework in aging research.

## Graphical abstract

Increased muscle entropy, measured by the homeostatic dysregulation index of muscle, reflects fiber disorganization. This accumulation of entropy independently contributes to declines in physical performance, muscle power, and mitochondrial energetics in older adults, establishing entropy as a key framework for skeletal muscle aging.

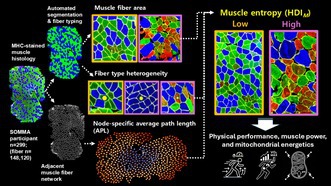

## 1 Introduction

The second law of thermodynamics specifies that chemical and physical structures become increasingly disorganized with time (Clausius 1865; Bortz 1986; Hershey and Lee 1987). At the biochemical level, such entropy in biological tissues arises from the random and irreversible rearrangement of molecules (e.g., protein aggregation) that disrupt the original structure and function (Pohl et al. 2012). Over time, these molecular disorders accumulate, undermining cellular and tissue organization and contributing to broader biological dysfunction (Gladyshev et al. 2021). Several cohorts have found associations between entropy, quantified by measurement of variability in the deviation from normal values in multiple blood chemistry markers, increased with age and predict adverse health outcomes including mortality, frailty, diabetes, and cardiovascular disease (Cohen et al. 2013, 2014; Li et al. 2015). The most common methods employed for quantifying entropy have been the homeostatic dysregulation index based on multivariable Mahalanobis distance and Shannon's information entropy (Mahalanobis 2018; Shannon 1948). It is unknown if these approaches are equivalent.

The arrangement of skeletal muscle fibers becomes disorganized with aging (Frontera et al. 2000, 2008; Kedlian et al. 2024). We postulate that the disordered structure is due to increasing entropy with aging. This possibility has not been studied. Furthermore, it is not known if increasing muscle entropy with aging is related to decreased skeletal muscle function and physical performance.

We used data and histology images from biopsies of the vastus lateralis in the Study of Muscle, Mobility and Aging (SOMMA), a cohort of community‐dwelling individuals age 70 years or older. We tested the hypothesis that greater entropy in the histologic characteristics of skeletal muscle fibers is associated with poorer performance on tests that involve muscle function, including 400‐m walk time, muscle power, peak oxygen uptake during cardiopulmonary exercise testing, short physical performance battery (SPPB), and mitochondrial energetics including maximal oxidative phosphorylation and ATP production in muscle mitochondria. We quantified entropy from fiber size diversity, fiber‐type heterogeneity, and spatial connectivity across fiber network. We also quantified Shannon Information entropy derived from variation of fiber size and tested its correlation with entropy quantified by the homeostatic dysregulation index.

## 2 Materials and Methods

### 2.1 Study Participants

SOMMA is a prospective cohort study of 879 participants aged 70 or older who were enrolled by field centers at University of Pittsburgh and Wake Forest University School of Medicine, from April 2019 to December 2021 (Cummings et al. 2023). The study was approved by the Western Institutional Review Board (20180764) and written informed consents were obtained from all participants. Individuals were excluded if they had any of the following: inability to walk ¼ mile or climb a flight of stairs, had BMI ≥ 40 kg/m^2^, a current malignancy, or advanced chronic illnesses (e.g., severe cardiac or pulmonary conditions that impair walking ability, end‐stage renal disease requiring dialysis, Parkinson's disease, or dementia). Individuals were excluded if they had a contraindication to having a muscle biopsy including taking anticoagulants, or a contraindication to Magnetic Resonance Spectroscopy including having ferrous metal‐containing implants.

### 2.2 Skeletal Muscle Biopsy, Sample Preparation, and Staining

Immunolabeling of myosin heavy chains (MHCs) to identify fiber types and muscle fiber morphology was performed in muscle cross‐sections cut from histology blocks of vastus lateralis muscle from 299 SOMMA participants who had muscle tissue with staining for histology. It is a convenient sample of the first participants to have adequate histology preparations. Briefly, 10 μm muscle cross‐sections were cut on a cryostat at −20°C, air‐dried for 5 min, and rinsed in PBST (0.05% Tween 20 in phosphate‐buffered saline). Slides were blocked in 10% goat serum in PBS for 1 h at room temperature, rinsed in PBST, and then incubated for 2 h at room temperature with a primary antibody against laminin (MAB1914P Millipore; 1:100) and primary MHC antibodies from the Developmental Studies Hybridoma Bank against BA‐F8 (type I MHC; 1:50), SC‐71 (type IIA; 1:500), and 6H1 (type IIX MHC; 1:25). Slides were washed three times for 10 min in PBS, allowed to dry, and then mounted with fluorescence mounting medium (Dako). Slides were kept at 4°C in the dark until they were imaged.

### 2.3 Muscle Fiber Segmentation, Fiber Typing, and Network Generation

Skeletal muscle histological sections stained for MHC isoforms were imaged using a fluorescence microscope (Olympus IX81) with a 20× objective (200× total magnification). Images were processed using Cellpose, a generalist algorithm for cellular segmentation, pretrained on cytoplasmic and tissue datasets (Stringer et al. 2021). A channel‐based postprocessing strategy was subsequently applied to optimize segmentation of muscle fibers, as detailed in the Supplementary Method. The model delineated individual muscle fiber boundaries based on MHC staining patterns. Following segmentation, fiber cross‐sectional area (CSA) was calculated for each fiber using pixel‐based area measurements. Fiber‐type classification (type I, IIA, IIX) was performed by quantifying fluorescence intensity of MHC isoform‐specific channels in the segmented muscle fiber masks. Fiber‐type heterogeneity was calculated as the number of neighboring fibers with a different fiber type than the index fiber, divided by the total number of adjacent fibers (range 0–1).

**FIGURE 1:**
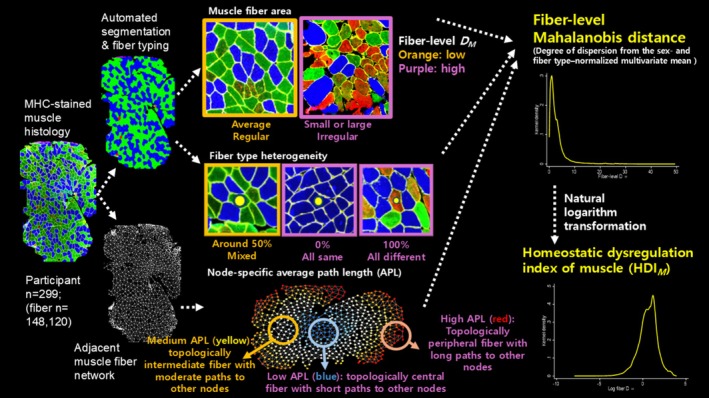
Analysis pipeline to calculate fiber‐level homeostatic dysregulation index of muscle (HDI_M_). In histology image panels, blue, green, and red fibers indicate type I, IIA, and IIx fiber, respectively. DM, Mahalanobis distance; APL, average path length.

To assess the spatial organization of muscle fibers, a two‐dimensional fiber adjacency network was constructed. In this network, each center point of muscle fiber was treated as a node, and edges were defined between directly adjacent fibers identified through shared segmented boundaries. Network topology was analyzed using Python (v3.9) and the NetworkX library. The node‐specific average path length (APL) assesses spatial connectivity of each node (fiber) (Watts and Strogatz 1998). For each muscle fiber, we computed the node‐specific APL, defined as the mean shortest path length from that fiber to all other reachable fibers within the fiber network. This metric yields a fiber‐level value. Both low‐ and high node‐specific APL values may indicate deviations either due to densely connected network by clustering of muscle fibers (topologically central) or dispersed network among muscle fibers, respectively (topologically peripheral). All analyses were conducted using custom Python scripts integrating outputs from Cellpose: automated image quantification workflows, and network topology (Figure 1).

### 2.4 Homeostatic Dysregulation Index of Muscle

Shannon entropy can only be calculated on a distribution and therefore cannot be calculated for individual muscle fibers. We therefore opted to use a homeostatic dysregulation index of muscle (HDI_*M*_) to calculate entropy at the fiber level. We used three histological features of muscle fibers: fiber size diversity, fiber‐type heterogeneity, and node‐specific APL of muscle fiber network to compute HDI_*M*_. These measure the deviation of a given muscle fiber from the mean of the normalized fiber characteristics for all participants. Prior to computing HDI_*M*_, all features were normalized using Z score at the fiber level after stratification by sex and fiber type (total six categories: two sex categories [women and men] × three fiber‐type categories [type I, type IIA, and type IIX]). For each normalized variable, the reference centroid was defined as zero, representing the mean of the normal distribution of fiber area, fiber‐type heterogeneity, and node‐specific APL in the fiber network. Mahalanobis distance was then calculated for each fiber based on the deviation of the three variables from its standardized centroids, using the covariance matrix derived from the entire fiber population, and defined as:

$$D_{M}=\sqrt{{\left(x-\mu \right)}^{Τ}S^{-1}\left(x-\mu \right)}$$

where x is the vector of normalized fiber‐level variables, μ is the reference centroid, and S is the covariance matrix estimated from the entire fiber population. Mahalanobis distance quantifies how far an observation deviates from the mean of a reference population, while considering the variance and covariance structure of the multiple variables (Mahalanobis 2018). Due to its pronounced right‐skewed distribution, the Mahalanobis distance was log‐transformed using the natural logarithm, without any offset, and the resulting value was defined as HDI_M_, a composite measure of muscle fiber‐level entropy (Cohen et al. 2013, 2014).

### 2.5 Correlation With Shannon Entropy

A correlation between HDI_M_ and Shannon entropy is not inherently guaranteed, as they measure different characteristics: statistical distance from the reference point and the informational uncertainty of the distribution, respectively (Shannon 1948). To empirically examine the potential relationship between these two metrics at the same analytical level, we implemented a subgrouping strategy at fiber level. All extracted fibers from the study population were first stratified according to similar values of HDI_M_ and grouped into 300 HDI_M_‐stratified fiber groups, with each group containing a comparable number of fibers (approximately 493–495 fibers per group). For each group, the median HDI_M_ value was used as the representative HDI_M_. Within each HDI_M_‐stratified fiber group, Shannon entropy was calculated separately for fiber CSA, fiber‐type heterogeneity index, and node‐specific APL, and was defined as:

$$H=-\sum_{i=1}^{k}p_{i}\log p_{i}$$

where $p_{i}$ is the probability of observations falling into the ith bin, and k is the total number of bins. For continuous variables, Shannon entropy estimation required discretization (Shannon 1948). The bin width was determined using the Freedman–Diaconis rule (Freedman and Diaconis 1981):

$$\text{bins}=2\cdot \frac{\mathrm{IQR}}{\sqrt[{3}]{n}}$$

where IQR is the interquartile range and n is the number of observations in the pooled fiber population. This bin width was then uniformly applied to all HDI_M_‐stratified fiber groups to ensure consistent and comparable Shannon entropy estimation across groups. Pearson correlation coefficients were computed between fiber group‐level HDI_M_ (median within each fiber group) and the Shannon entropy of each component.

### 2.6 Outcomes

Methods for SOMMA outcome collection were described previously (Cummings et al. 2023). The primary outcome of the study was 400‐m walk speed. The 400‐m walk test was performed at each participant's usual or preferred walking pace, consisting of 10 laps around a 40‐m course. The total time to complete the 400 m, recorded in seconds, included any rest periods taken during the test. Secondary outcomes include the expanded short physical performance battery (SPPB), leg power, grip strength, peak oxygen uptake (VO_2_ peak) during cardiopulmonary exercise testing on a treadmill, and mitochondrial energetics: including maximal oxidative phosphorylation and maximal ATP production. The expanded version of the SPPB, which includes a timed 4 m walk, chair stands, balance test, and short narrow walk, was measured (Simonsick et al. 2001). Leg extension power (W/kg) was assessed using the Keiser A420 Leg Press system. Grip strength was measured with a Jamar hand‐held dynamometer and a maximum of right and left grip strength was used. VO_2_ peak (mL/kg/min) was defined as the highest 30‐s average oxygen consumption recorded during the symptom‐limited, three‐stage cardiopulmonary exercise test (CPET) on a treadmill using either a modified Balke or manual protocol. Mitochondrial energetics were assessed using both ex vivo and in vivo approaches. High‐resolution respirometry was performed on permeabilized vastus lateralis fibers obtained via percutaneous biopsy to measure maximal oxidative phosphorylation (Max OXPHOS) supported by complex I‐ and II‐linked substrates (Coen et al. 2013; Mau et al. 2023). In vivo mitochondrial function was evaluated using ^31^P magnetic resonance spectroscopy (^31^P MRS), which estimates maximal ATP production (ATPmax) based on the rate of phosphocreatine recovery following knee extensor exercise.

### 2.7 Covariates

Weight was assessed by balance beam or digital scales, height by wall‐mounted stadiometers and BMI was calculated as weight (kg)/height (m^2^). Thigh muscle volume and total abdominal adipose tissue index (L/m^2^) were assessed using whole body magnetic resonance imaging (Cummings et al. 2023).

### 2.8 Statistical Analysis

Of the 879 older adults enrolled in the SOMMA project, we were able to obtain data on 299 older adults who had muscle histology tissue using data as of October 2025. Results are presented as mean ± standard deviation, median [interquartile range], or number (%). The mean CSA was only calculated among those who had proportion of corresponding fiber > 0%. Participant‐level HDI_*M*_ was defined as the mean of all fiber‐level HDI_*M*_ values within the corresponding muscle sample. In linear regression models, standardized β coefficients, representing the change in the outcome (in standard deviation units) per one standard deviation change in the predictor, were reported to allow comparison of effect sizes across variables measured on different scales. The figures illustrating the associations of HDI_*M*_ with physical performance and muscle measures present unadjusted standardized β coefficients derived from univariate models. We used two multivariable models to test the association between HDI_*M*_ and outcomes. Model 1 adjusted for age, sex, and BMI. For outcomes standardized for weight (leg power and VO_2_ peak), height was adjusted instead of BMI in model 1. Model 2 adjusted for age, sex, thigh muscle volume index (Z score, standardized to sex‐ and BMI‐matched virtual control groups) (Linge et al. 2020) and total abdominal adipose tissue index. We tested an interaction term for sex and the main predictor (HDI_*M*_) to assess whether the associations differ for women and men. Statistical significance threshold was set at two‐sided 0.05. All analyses were performed using Python 3.10, and Stata 18.0 (Statacorp, TX, USA).

## 3 Results

### 3.1 Characteristics of Study Participants

The mean age of these study participants was 76.4 ± 5.3 years, and 51.2% were women (Table 1, Appendix S1). We analyzed one MHC‐stained muscle histology image from each of the participants (Figure 1). A total of 148,120 fibers were segmented and quantified from the 299 samples (number of muscle fibers per sample: median 472, interquartile range 346–590). Type I fibers had the highest proportion (41.4%) and the largest average cross‐sectional area (CSA), followed by type IIA and type IIX fibers. Approximately half of the neighboring fibers (49% ± 10%) differed in fiber type from the index fiber. The mean node‐specific APL of the fiber network was 13.3 ± 3.4.

**TABLE 1:** Clinical characteristics of study participant (*n* = 299).

Clinical characteristics	Values
Age, year	76.4 ± 5.3
Women, *n* (%)	153 (51.2)
Body mass index, kg/m^2^	27.9 ± 4.8
400‐m walk speed, m/s	1.0 ± 0.2
Expanded SPPB score (*n* = 298)	2.1 ± 0.5
VO_2_ peak, mL/kg/min (*n* = 279)	20.2 ± 4.4
Peak leg power (weight‐standardized), Watts/kg (*n* = 287)	4.8 ± 1.8
Grip strength, kg	30.2 ± 10.1
Max OXPHOS, pmol/(s*mg) (*n* = 288)	57.83 ± 17.04
ATP_MAX_, mM/s (*n* = 276)	0.55 ± 0.15
Thigh muscle volume, L (*n* = 280)	9.2 ± 2.4
Thigh muscle volume, Z score (*n* = 280)	−0.8 ± 1.0
Total abdominal adipose tissue index, L/m^2^ (*n* = 284)	4.4 ± 1.7
Skeletal muscle histology parameters	
Number of muscle fibers per participant	472 [346–590]
Average muscle fiber CSA, μm^2^	
Type I	4535 [3461–5543]
Type IIA	3134 [2161–4454]
Type IIX	2238 [1394–3466]
Proportion of fiber types, %	
Type I	41.4 [30.6–51.2]
Type IIA	34.6 [25.7–45.4]
Type IIX	0.3 [0.0–3.1]
Node‐specific average path length	13.3 ± 3.4
Fiber‐type heterogeneity	0.49 ± 0.10

*Note:* Data were presented as mean ± standard deviation, median [interquartile range], or number (%). The mean CSA was only calculated among those who had proportion of corresponding fiber > 0%.

Abbreviations: ATP_MAX_, maximal ATP production; CSA, cross‐sectional area; Max OXPHOS, maximal oxidative phosphorylation; SPPB, short physical performance battery; VO_2_ peak, peak oxygen consumption.

### 3.2 Homeostatic Dysregulation Index of Muscle (HDI_M_ )

**FIGURE 2:**
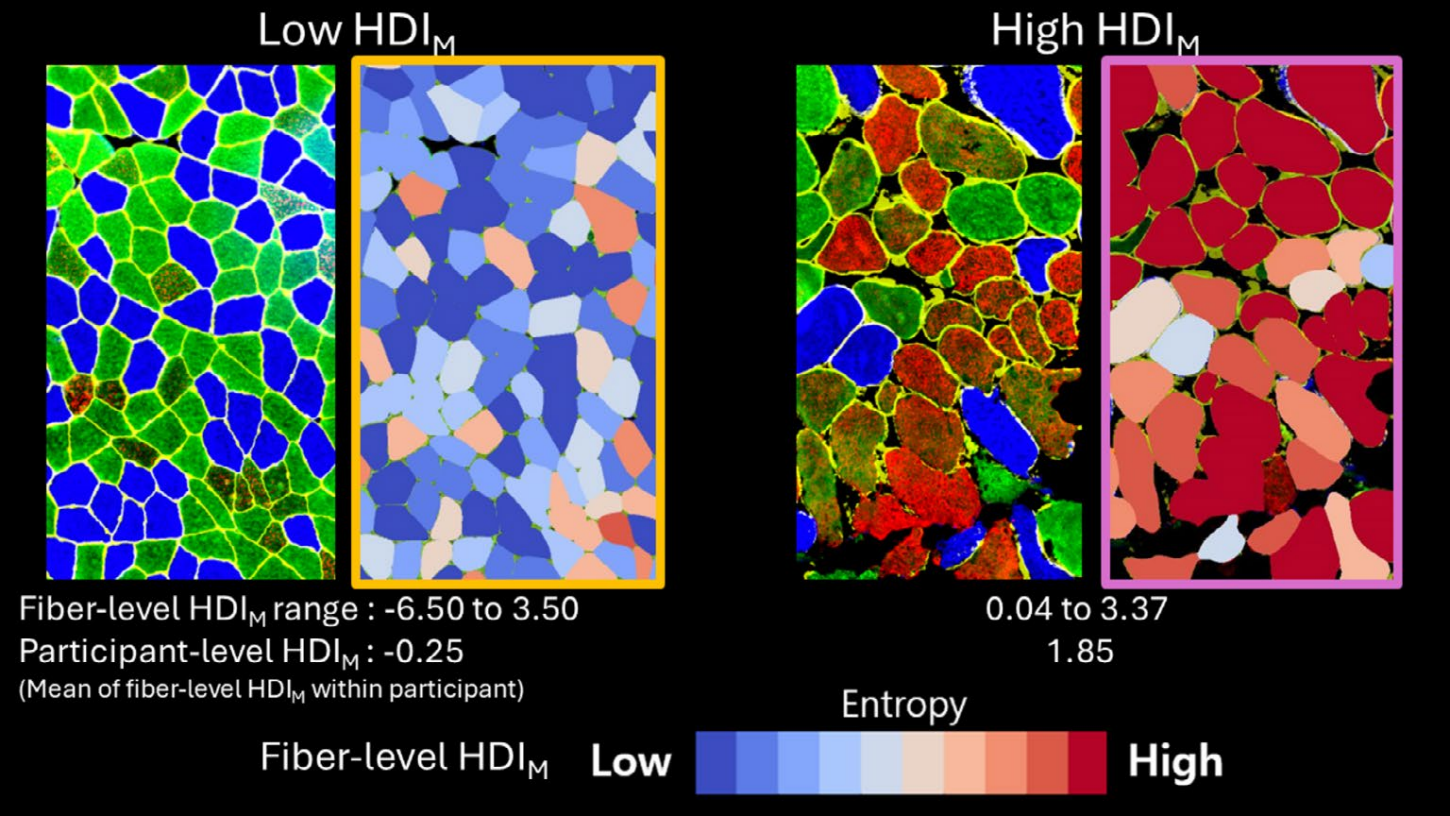
Graphical examples of histology images with low and high homeostatic dysregulation index of muscle (HDI_M_) as a measure of entropy. In the MHC‐stained histology images (unboxed images), blue, green, and red fibers indicate type I, IIA, and IIx fiber, respectively. In images mapped according to deciles of HDI_M_ values (boxed images), more blue indicates lower HDI_M_ value in decile, whereas more red indicates higher HDI_M_ value. Fiber‐level HDI_M_ values shown here are subsequently aggregated to derive participant‐level HDI_M_.

HDI_M_ was defined as the natural log‐transformed Mahalanobis distance derived from fiber CSA, fiber‐type heterogeneity, and node‐specific average path length, relative to the sex‐ and fiber‐type‐normalized global mean (see Materials and Methods for details). Mean fiber‐level HDI_*M*_ value was 0.58 ± 1.08. Figure 2 shows fibers with lower HDI_*M*_ values in blue and higher values in red, with gradation reflecting intermediate values. Greater deviation from the average in any of the muscle histological characteristics results in a higher HDI_*M*_ value. In Figure 2, the left panel illustrates predominantly low fiber‐level HDI_*M*_ values, corresponding to a low participant‐level HDI_*M*_ (average of fiber‐level HDI_*M*_ values), whereas the right panel shows more fibers with intermediate to high values, resulting in a higher participant‐level HDI_*M*_.

### 3.3 HDI_M_ and Shannon Entropy (Information Entropy)

**FIGURE 3:**
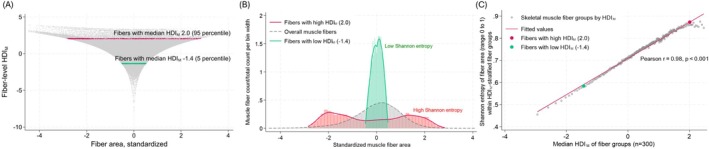
Correlation between homeostatic dysregulation index of muscle (HDI_M_) and information entropy (Shannon entropy, standardized; range 0–1) of each component of HDI_M_ (fiber area, node‐specific average path length, and fiber‐type heterogeneity) with HDI_M_‐stratified fiber groups. (A) y‐axis represents fiber‐level HDI_M_ values for all fibers (*n* = 148,120), and x‐axis represents standardized (Z score) fiber area. Fibers with low HDIM values were closely clustered around 0. As fiber‐level HDI_M_ values increase, the distribution of fiber area is becoming widely scattered, showing increased uncertainty (increased entropy). (B) For example, muscle fibers with high HDI_M_ (2.0, red; 95 percentile among fiber groups) showed wider dispersion of standardized fiber area distribution (high uncertainty = high Shannon entropy: 0.87), whereas fibers with low HDI_M_ value (−1.4, green; 5 percentile) have narrow muscle fiber area distribution around the population average (low uncertainty = low Shannon entropy: 0.57). (C) To test whether having high HDI_M_ (more deviation from the norm) values are correlated with more uncertainty of fiber characteristics (greater dispersion in distribution), fibers were stratified into 300 groups (493–495 fibers per each fiber group) by HDI_M_ values. The median HDI_M_ was used as represenatative value for each fiber group. At HDI_M_‐stratified fiber group level, median HDI_M_ of fiber groups and Shannon entropy of fiber area calculated within each fiber group showed strong positive correlation (Pearson correlation coefficient 0.98, *p* < 0.001). X‐axis indicates median HDI_M_ value as representative value for each fiber group. Note that the red and green dots are the fiber groups shown in parts A and B.

We hypothesized that fibers with higher HDI_M_ values would exhibit more disordered and unpredictable characteristics, such as area, compared to those with low HDI_M_. To test this, muscle fibers were stratified based on their HDI_M_ scores into 300 groups (493–495 fibers per group; median HDI_M_ as representative value for a group). Shannon entropy of fiber area, node‐specific APL, and fiber‐type heterogeneity was calculated for fiber groups to quantify the degree of structural uncertainty. We performed a correlation analysis to determine whether statistical deviation from the norm at the fiber group‐level (HDI_M_) is systematically associated with structural uncertainty (Shannon entropy) within the group. Fibers with higher HDI_M_ values are associated with a greater spread in the distribution of individual fiber characteristics, indicating an increased structural uncertainty (Figure 3A). In Figure 3B, a histogram tightly clustered around the mean (green; 5 percentile) indicates low variability in fiber group distribution, suggesting a more uniform or ordered state (low Shannon entropy). In contrast, a widely dispersed histogram reflects greater variability and randomness in fiber distribution (red; 95 percentile), consistent with higher Shannon entropy. Consistent with our hypothesis, the median HDI_M_ of fiber groups demonstrated a strong positive correlation with the Shannon entropy of fiber area within each group (Pearson *r* = 0.98; Figure 3C). Similar robust associations were observed for node‐specific APL (*r* = 0.99) and fiber‐type heterogeneity (*r* = 0.68; Appendix S1). To further validate these findings, we examined the correlation between fiber group‐level‐modified HDI_M_ (calculated excluding area information) and the Shannon entropy of fiber area, which yielded a significant, though more modest, positive correlation (*r* = 0.53), confirming the robustness of the observed relationship (Appendix S1).

### 3.4 Associations Between HDI_M_ and Outcomes

**FIGURE 4:**
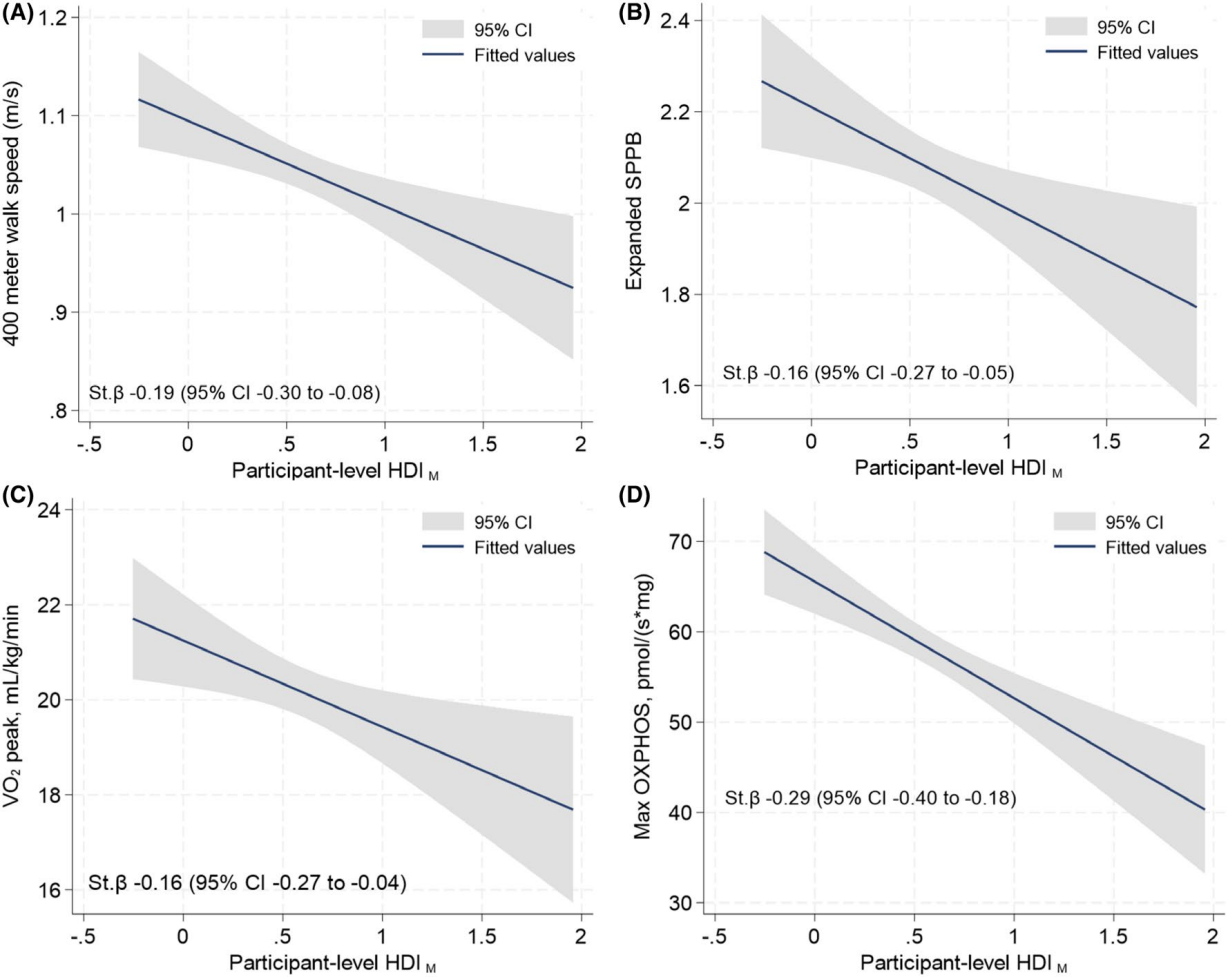
Association of participant‐level homeostatic dysregulation index of muscle (HDI_M_) with (A) 400‐m walk speed (m/s), (B) expanded short physical performance battery (SPPB), (C) peak oxygen consumption (peak VO_2_), and (D) maximal oxidative phosphorylation (max OXPHOS). St.β, unadjusted standardized beta coefficient derived from univariate linear regression models.

No statistically significant interaction between HDI_*M*_ and sex was observed for any outcomes (*p* for interaction > 0.11 for all; data not shown). When the fiber‐level HDI_M_ within a participant was used as participant‐level HDI_M_ value, we found no significant association between participant‐level HDI_M_ and age in a linear regression model (β = 0.001, 95% CI −0.007 to 0.008). Greater participant‐level HDI_*M*_ was associated with a statistically significant lower 400‐m walk speed even after adjustment for age, sex, and BMI (model 1) and after further adjustment for age, sex, thigh muscle volume index, and total adipose tissue index (model 2; Table 2). HDI_*M*_ had stronger associations with outcomes than any of its components (fiber area, APSL, and fiber‐type heterogeneity; Appendix S1). In univariate and multivariable‐adjusted models, greater HDI_*M*_ was associated with poorer performance on the SPPB, lower grip strength, lower leg power, lower oxidative phosphorylation (Max OXPHOS), and lower ATP_max_ (Figure 4, Table 2).

**TABLE 2:** Association of participant‐level homeostatic dysregulation index of muscle (HDI_*M*_) with physical performance, muscle power, strength, and mitochondrial energetics.

Outcomes	Model 1 (age, sex, and BMI adjusted)		Model 2 (age, sex, muscle index, and total adipose tissue index adjusted)	
	Standardized β coefficient (95% CI)	*p*	Standardized β coefficient (95% CI)	*p*
400‐m walk speed	−0.16 (−0.26 to −0.06)	0.002	−0.17 (−0.28 to −0.06)	0.002
Expanded SPPB	−0.14 (−0.24 to −0.04)	0.008	−0.16 (−0.27 to −0.06)	0.003
VO_2_ peak*	−0.16 (−0.26 to −0.05)	0.003	−0.17 (−0.26 to −0.08)	< 0.001
Grip strength	−0.09 (−0.16 to −0.01)	0.028	−0.10 (−0.19 to −0.02)	0.012
Standardized leg power*	−0.10 (−0.20 to −0.01)	0.030	−0.12 (−0.21 to −0.03)	0.009
Max OXPHOS	−0.27 (−0.38 to −0.17)	< 0.001	−0.29 (−0.39 to −0.18)	< 0.001
ATP_max_	−0.15 (−0.27 to −0.03)	0.011	−0.16 (−0.28 to −0.03)	0.013

*Note:* Standardized β coefficients represent the change in the dependent variable (in standard deviation units) per one standard deviation increase in the independent variable. *For outcomes standardized for weight (leg power, VO_2_ peak), height was adjusted instead of BMI in Model 1.

Abbreviations: ATP_MAX_, maximal ATP production; CSA, cross‐sectional area; Max OXPHOS, maximal oxidative phosphorylation; SPPB, short physical performance battery; VO_2_ peak, peak oxygen consumption.

## 4 Discussion

Our findings support the hypothesis that muscle fibers become disorganized in a way that can be quantified as entropy that increases with age. Furthermore, greater fiber entropy measured using three characteristics of muscle fibers, including fiber cross‐sectional area, fiber‐type heterogeneity, and node‐specific APL of the fiber network, was strongly associated with slower 400‐m walk speed, poorer performance on the SPPB, weaker muscle power and grip strength, lower VO_2_ peak, and reduced mitochondrial energetics including maximal oxidative phosphorylation and maximal ATP production.

Entropy of physical substances increases with time (Clausius 1865). HDI_*M*_ derived from three skeletal muscle histology characteristics behaved as expected for a measurement of entropy. HDI_*M*_ correlated highly with another measurement of entropy, Shannon's information entropy. Most importantly, as hypothesized, increasing HDI_*M*_ was associated with several measurements of poorer muscle function and physical performance measurements.

Aging skeletal muscle exhibits progressive histological disorganization characterized by fiber size heterogeneity, where both small atrophic and large hypertrophic fibers coexist (Scelsi et al. 1980; Andersen 2003; Lexell and Taylor 1991). Additional changes include fiber‐type grouping and increased co‐expression of multiple MHC isoforms within individual fibers and may contribute to disorganization and other processes occurring in aging muscle (Andersen 2003). For example, these changes could result from cycles of denervation and reinnervation, leading to sporadic atrophy, compensatory hypertrophy across motor units, accumulation of small angular fibers, and clustering of fibers with the same MHC expression (Rowan et al. 2011, 2012). Some changes may result from insufficient physical exercise or activity, adiposity, or other factors (Tøien et al. 2023). Collectively, these features represent a gradual loss of structural integrity in skeletal muscle (Hepple 2012). Conceptualizing these changes as histological entropy provides a unifying framework for understanding the complex disorganization of muscle. Quantification of entropy in skeletal muscle histology images could serve as a biomarker capturing several morphological changes and the degree of structural disorganization. This measure may also help track longitudinal changes in response to interventions targeting muscle aging.

These findings are important beyond muscle. Stochastic accumulation of errors and damage across biological scales is increasingly thought to be the main force of aging of living organisms. These specific entropies, such as DNA damage and tissue disorganization, eventually contribute through feedback loops to increased system‐wide entropy (Meyer et al. 2025). Thus, entropy is becoming an important unifying framework in aging epidemiology. Studies of blood‐based biomarkers have consistently demonstrated that HDI, a marker of physiological dysregulation, rises with age and is a strong predictor of negative health outcomes such as mortality, frailty, diabetes, and cardiovascular disease (Cohen et al. 2013, 2014; Li et al. 2015). Our work here shows that HDI can be considered a metric of entropy, allowing us to reframe these previous results, which apply in many systems and integrate across them. As another example, we recently showed that entropy of the cardiac conduction system, measured by HDI of electrocardiogram (ECG) parameters, increased with age and was predictive of fracture and mortality in a large cohort (Hong et al. 2025). The prediction of outcomes prima facie unrelated to ECG supports the integrated nature of entropy across biological systems. The method used to define entropy of muscle in this study is widely applicable to various levels of measurements including blood biomarkers, images, and multi‐omics profiles, providing opportunities for extensive validation and extension of the entropy framework. An important conceptual question is whether the entropy quantified here reflects muscle‐specific disorganization or a broader, organism‐level aging process. While HDI_M_ is derived from muscle fiber‐level features and therefore captures the degree of disorganization of local tissue, it may also be interpreted as a sentinel marker of systemic aging, potentially reflecting progressive exhaustion of compensatory and homeostatic mechanisms with aging. Although direct evaluation of multisystem aging constructs was beyond the scope of this study, this is an important direction for future research and requires accumulation of integrated, multisystem data.

Mahalanobis distance and Shannon information entropy are different approaches to quantifying entropy that have different applications (Cohen et al. 2013, 2014; Li et al. 2015; Byun et al. 2019). Information entropy requires that multiple measurements exist within an individual (Shannon 1948). In contrast, Mahalanobis distance quantifies how far an individual deviates from a reference population, even with a single value per individual. We found that HDI_*M*_ calculated using Mahalanobis distance is highly correlated with information entropy across individual fiber traits. This finding indicates that both methods are assessing the underlying concept of entropy and supports the use of HDI_*M*_ as a valid proxy for entropy.

This study has limitations. Entropy is a complex phenomenon that spans scales from the molecular to the tissue and whole‐organism levels, making it inherently challenging to define and quantify since it captures the randomness or disorganization in biological systems (Bortz 1986). Muscle samples from study participants aged 70 or older were analyzed in this study and all the participants would be expected to exhibit a degree of deviation in HDI_*M*_ from a reference young population. This is a cross‐sectional study and changes with age should be confirmed in longitudinal follow‐up. Some factors that change with age, such as a decrease in a variety of activity, muscle function, factors associated with decreased muscle mass, body composition, or other factors, might also contribute to an increase in entropy with age which may be explored in other analyses. The absence of an association between participant‐level HDI_*M*_ and age may reflect information loss from aggregating fiber‐level HDI_*M*_ into a single mean per participant, which obscures within‐participant heterogeneity. In addition, the restricted age range of the SOMMA cohort may have limited our ability to detect age‐related gradients. The trajectory of HDI_*M*_ across a wider age span needs to be investigated. Future studies comparing the predictive performance of participant‐level HDI_M_ with simpler Shannon entropy‐based metrics would be valuable for both mechanistic insight and broader implementation. This study did not explicitly account for mixed muscle fiber types (e.g., MHC I/IIA and IIAX), which may have led to an underestimation of fiber‐type complexity.

In summary, we conclude that skeletal muscle fibers undergo disorganization that can be characterized as entropy. HDI_*M*_ increased with age and correlated with physical performance, peak oxygen consumption during cardiopulmonary exercise, muscle power and maximum oxidative phosphorylation of muscle, independent of thigh muscle volume. This approach can be applied to other data and images to test that entropy increases with aging and this natural physical process is associated with loss of functions. Whether development of entropy in histological traits of skeletal muscle can be slowed, for example, by resistance training, remains to be determined.

## Author Contributions

**Namki Hong:** conceptualization, methodology, formal analysis, investigation, data curation, visualization, writing – original draft, writing – review and editing; **Sang Wouk Cho:** software, methodology, formal analysis, investigation, visualization, writing – original draft, writing – review and editing; **Alan A. Cohen:** conceptualization, methodology, resources, writing – review and editing; **Russell T. Hepple:** methodology, validation, data curation, writing – review and editing, resources; **Paul M. Coen:** writing – review and editing, resources, data curation; **Bumsoo Ahn:** writing – review and editing, resources; Anne B. Newman: writing – review and editing, resources; **Stephen B. Kritchesky:** writing – review and editing, resources; **Paul J. Laurenti:** validation, writing – review and editing; **Warren S. Browner:** writing – review and editing; **Steven R. Cummings:** conceptualization, methodology, project administration, funding acquisition, investigation, resources, writing – original draft, writing – review and editing. All authors have read and finally agreed to the published version of the manuscript.

## Funding

This work was supported by the grant of the National Institute on Aging (NIA grant number R01AG059416).

## Conflicts of Interest

The authors declare no conflicts of interest.

## Supporting information


**Appendix S1:** acel70421‐sup‐0001‐AppendixS1.pdf.

## Data Availability

The data that support the findings of this study are openly available in SOMMA Online at https://sommaonline.ucsf.edu/.
