# Supplementary material for: Entropy of Muscle Fiber Histology Predicts Mobility in Older Adults: The Study of Muscle, Mobility, and Aging
Source: Aging Cell. 2026 Feb 22;25(3):e70421. doi: 10.1111/acel.70421 (PMC12927989; doi:10.1111/acel.70421)
Supplement: Supplementary file 1 — Appendix S1: acel70421‐sup‐0001‐AppendixS1.pdf. [file ACEL-25-e70421-s001.pdf]

(Supporting Information) Entropy of muscle fiber histology predicts mobility in older adults: The Study of Muscle, Mobility and Aging

Short title: Muscle entropy and mobility

Namki Hong*[1,2,3], Sang Wouk Cho*[4], Alan A. Cohen[5], Russell T Hepple[6], Paul M Coen[7], Bumsoo Ahn[8], Anne B. Newman[9], Stephen B. Kritchesky[10], Paul J. Laurenti[11], Warren S. Browner[1], and Steven R. Cummings[1,2]

1. San Francisco Coordinating Center, California Pacific Medical Center Research Institute, San Francisco, CA, USA
2. Department of Epidemiology and Biostatistics, University of California, San Francisco, San Francisco, CA, USA
3. Department of Internal Medicine, Institute of Endocrine Research, Yonsei University College of Medicine, Seoul, South Korea
4. Department of Biomedical Systems Informatics, Yonsei University, College of Medicine, Seoul, South Korea
5. Department of Environmental Health Sciences, Butler Columbia Aging Center, Mailman School of Public Health, Columbia University, New York, NY, USA
6. Department of Physical Therapy, University of Florida, Gainesville, Florida
7. AdventHealth, Translational Research Institute, Orlando, FL USA
8. Department of Internal Medicine, Wake Forest University School of Medicine, Winston-Salem, North Carolina
9. Department of Epidemiology, School of Public Health, University of Pittsburgh, Pittsburgh, Pennsylvania
10. Department of Internal Medicine, Section on Gerontology & Geriatric Medicine and the Sticht Center for Healthy aging and Alzheimer’s Prevention, Wake Forest University School of Medicine, Winston-Salem, North Carolina
11. Department of Radiology, Wake Forest University School of Medicine, Winston-Salem, North Carolina

*These authors contributed equally to this work.

Corresponding author: Steven R. Cummings

Steven.cummings@ucsf.edu; 415-203-2864

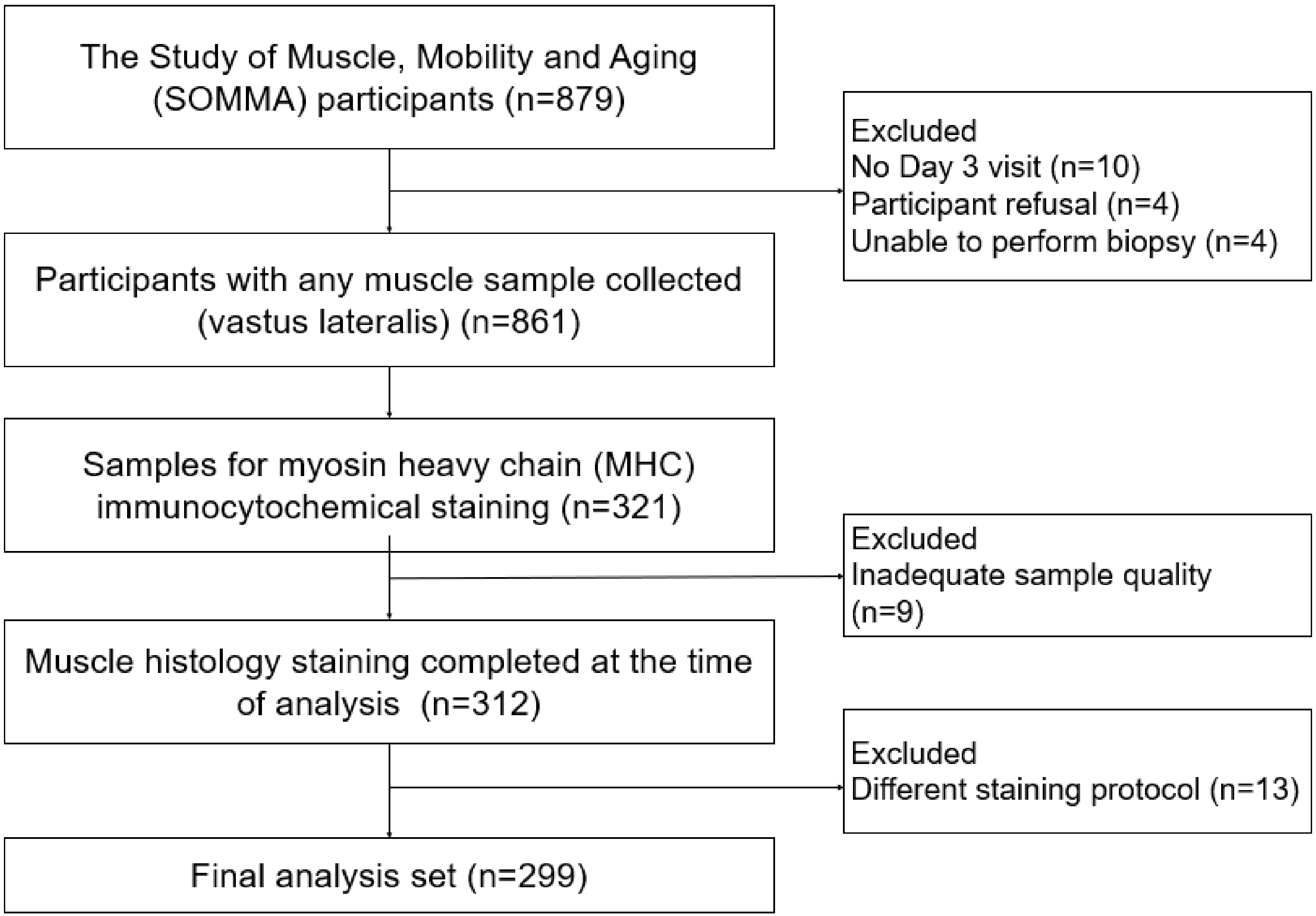

Supplementary Figure 1. Study flow

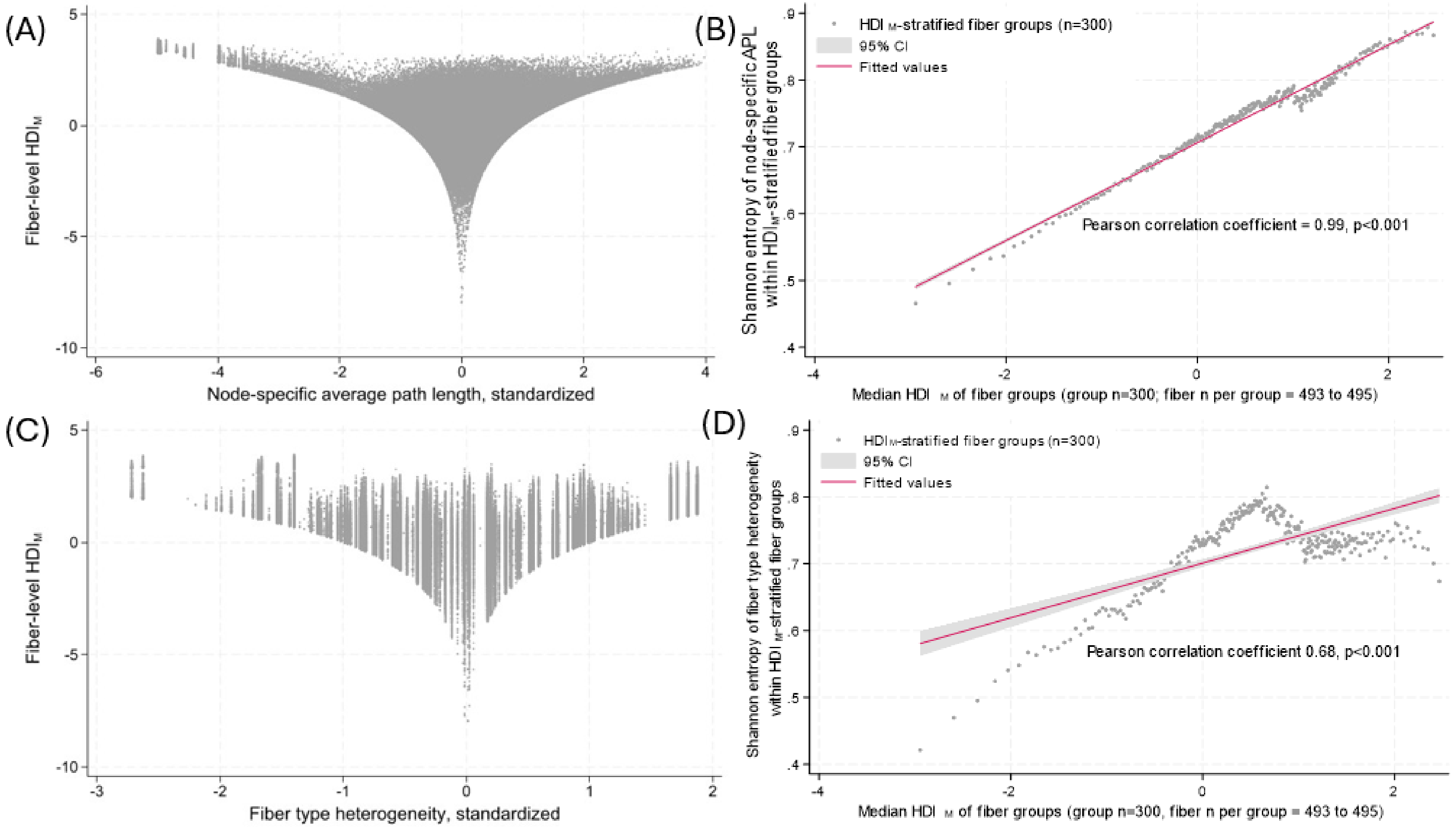

Supplementary figure 2. (A) Relationship between fiber-level homeostatic dysregulation index of muscle (HDI_M) and standardized (Z-score) node-specific average path length (APL). (B) Correlation between Shannon entropy of node-specific ASPL and median HDI_M across HDI_M-stratified fiber groups. (C) Relationship between fiber-level HDI_M and standardized fiber type heterogeneity (standardized, Z-score). (D) Correlation between Shannon entropy of fiber type heterogeneity and median HDI_M across HDI_M-stratified fiber groups.

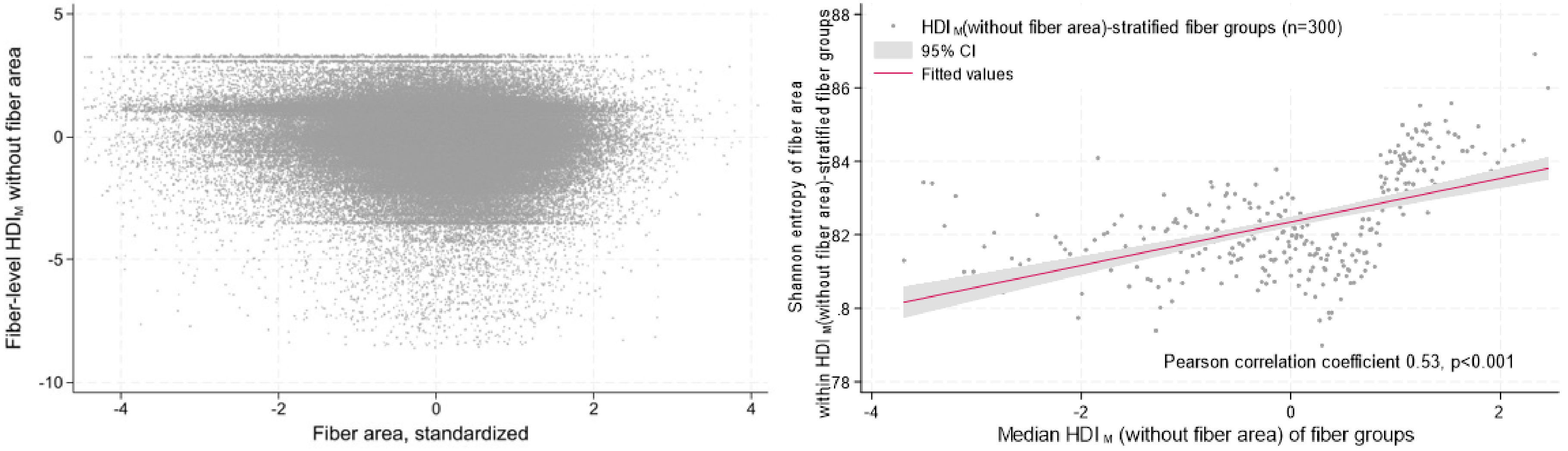

Supplementary figure 3. (A) Relationship between fiber-level homeostatic dysregulation index of muscle (HDI_M excluding fiber area information) and standardized (Z-score) fiber area. (B) Correlation between Shannon entropy of fiber area and median HDI_M (excluding fiber area information) across HDI_M (excluding fiber area information)-stratified fiber groups.

Supplementary table 1. Association of participant-level homeostatic dysregulation index of muscle, muscle fiber CSA, node-specific APL, fiber type heterogeneity with 400 m walk speed in multivariable linear regression model

Predictors	Model 1 (age, sex, and BMI adjusted)		Model 2 (age, sex, muscle index, and total adipose tissue index adjusted)	
	Standardized beta coefficient (95% CI)	P-value	Standardized beta coefficient (95% CI)	P-value
HDI_M	-0.16 (-0.26 to -0.06)	0.002	-0.17 (-0.28 to -0.06)	0.002
Muscle fiber CSA	0.10 (-0.02 to 0.23)	0.096	0.04 (-0.09 to 0.17)	0.571
Node-specific APL	-0.12 (-0.23 to -0.02)	0.024	-0.09 (-0.20 to 0.03)	0.152
Fiber type heterogeneity	-0.03 (-0.13 to 0.07)	0.571	-0.05 (-0.15 to 0.06)	0.366

Each predictor entered the model separately. Standardized beta coefficients represent the change in the dependent variable (in standard deviation units) per one standard deviation increase in the independent variable. Participant-level muscle fiber CSA, ASPL, and fiber type heterogeneity index was calculated as the average of corresponding values of skeletal muscle fibers in each participant. Abbreviations: HDI_M, homeostatic dysregulation index of muscle; CSA, cross-sectional area; node-specific APL, average path length.

Supplementary method

Post-processing to improve cellpose performance with multi-channel segmentation strategy

A multi-channel segmentation and center-consensus post-processing was adopted to improve performance of cellpose algorithm. Segmentation was performed independently on four channels: Red (R), Green (G), Blue (B), and Gray-scale images derived from the original RGB input.

1. Mask and center extraction: for each channel (R, G, B, and gray), cellpose algorithm was applied independently to generate instance masks, from which object center points were computed.

2. Gray-anchored center validation: a center point detected in the Gray-channel was retained if it lay within a 5-pixel radius of a center point detected in at least one of the R, G, or B channels. This step was designed to ensure that candidate objects were supported by both intensity-aggregated (gray) and color-specific information.

3. Resolution of overlapping color-channel detections: when overlapping center points were detected between the R and G channels, the R channel center point was retained if it was more than 10 pixels away from the Gray-validated centers identified in step 2. This rule reduced redundant detections while preserving channel-specific sensitivity.

This strategy was motivated by the complementary nature of grayscale and color-specific information. The Gray channel provides stable structural cues by integrating intensity across channels, while individual RGB channels capture channel-specific contrast variations that may be suppressed in grayscale conversion.
